# Draft Genome Sequence of *Rhodococcus* sp. Strain M8, Which Can Degrade a Broad Range of Nitriles

**DOI:** 10.1128/genomeA.01526-17

**Published:** 2018-02-08

**Authors:** Andrey D. Novikov, Konstantin V. Lavrov, Artem S. Kasianov, Tatyana V. Gerasimova, Alexander S. Yanenko

**Affiliations:** a State Research Institute for Genetics and Selection of Industrial Microorganisms, Moscow, Russia; b Vavilov Institute of General Genetics, Moscow, Russia

## Abstract

*Rhodococcus* sp. strain M8 is a nitrile-degrading bacterium isolated from acrylonitrile-contaminated sites. This strain produces the enzymes for sequential nitrile degradation, cobalt-type nitrile hydratase, and amidase in large amounts. Its draft genome sequence, announced here, has an estimated size of 6.3 Mbp.

## GENOME ANNOUNCEMENT

Synthetic nitriles are widely employed in organic synthesis as precursors of various amides and acids, which are obtained via hydration of the triple C-N bond in the nitriles (1, 2). One of the most important processes is acrylonitrile transformation to acrylamide and acrylic acid, which are indispensable monomers for polymer chemistry. The perspective method of nitrile transformation is biocatalytic hydrolysis using the nitrile-hydrolyzing enzymes nitrile hydratases (NHases) and nitrilases. Contrary to conventional chemical hydrolysis, enzymatic processes take place under mild conditions (neutral pH and room temperature), selectively involve only nitrile groups, and are in some cases stereoselective, which is significant in the synthesis of biologically active compounds (3).

*Rhodococcus* sp. M8, a strain isolated from acrylonitrile-contaminated soil, can transform and utilize a broad range of nitriles (acrylonitrile, acetonitrile, propionitrile, and benzonitrile [4]). The highly efficient cobalt-activated expression of cobalt-type NHase and aliphatic amidase in it was the first discovered example of metal-dependent regulation of NHase cluster expression in bacteria (5–8).

The strain was grown in mineral salt medium at 30°C (5). The genomic DNA was prepared using the phenol-chloroform method and sequenced using the Illumina HiSeq 2500 platform by Genotek (Moscow, Russia). The sequencing generated paired-end reads of 100 bp, which were assembled using SPAdes version 3.9 (9), with the “careful” option, producing 163 scaffolds with a total genome size of 6,243,964 bp (70% GC content; *N*_50_, 221.09 kbp) and an average 54-fold coverage. The genome was also resequenced using PacBio and assembled using a standard Pacific Biosciences pipeline. Here, we present the genome sequence of *Rhodococcus* sp. M8, obtained using Illumina and PacBio technologies, containing 6 scaffolds, which cover approximately 99.5% of the genome. Automatic annotation was performed using the PROKKA pipeline version 1.11 (10), generating 5,753 features potentially assigned to protein-coding genes. The genome has a total size of 6,317,587 bp (68% GC content; *N*_50_, 3,396.772 kbp) and contains 55 tRNA genes, 1 transfer-messenger RNA (tmRNA) gene, and 3 rRNA operons. An analysis of similarity among the *Rhodococcus* genomes, measured as average nucleotide identity (ANI), shows that the genome of the *Rhodococcus* sp. M8 is most similar to that of *Rhodococcus aetherivorans* (GenBank accession no. CP011341, 89% similarity).

The genome contains two different NHase clusters. The first contains the cobalt-type NHase gene *nhmBA* with its regulatory and maturation-helping genes *nhmCDG* (GenBank accession no. AY654301), similar to the Co-type H-NHase cluster from *Rhodococcus rhodochrous* J1 (GenBank accession no. D67027). The second contains Fe-type NHase, its regulatory gene, phenylacetaldoxime dehydratase, and amidase genes, similar to the corresponding genes of the Fe-type NHase cluster from *Rhodococcus erythropolis* (GenBank accession no. AB016078). There are no any known nitrilase genes in this strain.

Extensive genome analysis of this and other NHase-producing *Rhodococcus* strains is of fundamental and practical interest due to poor knowledge about *Rhodococcus* genetics and an increasing number of biotechnological applications of *Rhodococcus* strains.

### Accession number(s).

This whole-genome shotgun sequence has been deposited at GenBank/DDBJ/EMBL under the accession no. MLYX00000000. The version described in this paper is the second version, MLYX02000000.
